# A Study on the Detent Torque and Holding Torque of a Micro-Claw Pole Stepper Motor

**DOI:** 10.3390/mi13060931

**Published:** 2022-06-11

**Authors:** Xiaofei Xi, Yan Sun, Xudong Wang, Yuanxu Xin, Yong Yang

**Affiliations:** School of Electrical Engineering, Shanghai Dianji University, Shanghai 201306, China; 206001010408@st.sdju.edu.cn (X.X.); 206001010223@st.sdju.edu.cn (Y.X.); 206001010311@st.sdju.edu.cn (Y.Y.)

**Keywords:** micro motor, claw pole stepper motor, detent torque, Taguchi experiment

## Abstract

The micro-claw pole stepper motor is widely used in the field of camera modules and VR focusing. The influence of torque ripple on positioning accuracy becomes more obvious with a decrease in motor volume. In order to reduce the torque ripple of the micro-claw stepper motor and increase the load capacity of the motor, the torque of the motor is simulated by using finite element software. Firstly, the influences of four parameters, namely air gap, magnet thickness, claw thickness and claw height, on the detent torque and holding torque of the claw permanent magnet stepper motor are obtained through the Taguchi experiment. The Signal-to-noise ratio (SNR) of each factor to the response was calculated and the degree of influence of the four parameters on the detent torque and holding torque of the micro-claw pole permanent magnet stepper motor was determined. Then, the optimal value of each factor to reduce the detent torque and increase the holding torque was obtained through optimization analysis. Finally, experiments were carried out to test the holding torque of the motor, and the accuracy of the results was verified by comparing the test values with the simulation values. According to the analysis of the paper, the response delta of air gap to detent torque is the largest, reaching 5.99, and that to holding torque is 0.73. The response delta of the magnet thickness to the detent torque is 5.87, and the response delta to the holding torque is 1.52. The optimized parameters obtained by optimization analysis reduce the detent torque of the motor by 26.74% and increase the holding torque by 18.35%. It is found that air gap and permanent magnet thickness have the greatest influence on the detent torque and holding torque of a micro-claw permanent magnet stepper motor, followed by claw thickness and claw height. Among them, the air gap has more influence on the detent torque than on the holding torque, and the thickness of the permanent magnet has more influence on the holding torque than on the detent torque.

## 1. Introduction

The micro-claw pole permanent magnet stepper motor has the advantages of small size, easy control and low cost [1]. In recent years, it has been widely used in the field of the focus adjustment of mobile phone lenses and virtual reality (VR) devices. In the driving system of the camera module, the stepping motor realizes the focal length adjustment of the lens through the precise screw drive [2]. However, the inherent existence of its detent torque affects the stability and positioning accuracy of the motor [3]. As the size of the permanent magnet stepper motor becomes smaller and smaller, the side effect of its detent torque increases relatively. When the positioning torque is too large, it results in a series of problems such as high power consumption, noise and inaccurate focusing positioning.

Therefore, scholars are mainly interested in the static and dynamic torque characteristics of motors [4,5,6,7,8,9,10]. The design of the control algorithm or drive circuit can improve the torque characteristics of the motor and improve the positioning accuracy [11,12], but the improvement effect of the control algorithm is limited. Hence, the motor body parameters become the main goal of analyzing and optimizing the motor performance. There have been many studies using 3D finite element simulation [13,14,15,16,17,18]. Through finite element analysis, the influences of different permanent magnet thickness combinations on the working point, air gap flux density, noload and load characteristics of the magnet are compared. It is concluded that NdFeB can improve the air-gap flux density on the one hand and resist the influence of armature reaction on the other hand, but when the magnet is too thick, the air-gap flux density will be difficult to adjust [19]. The permanent magnet shape of the motor is also optimized in [20]. The authors of [21] analyzed the air gap of the motor for optimum torque production in a given volume. In [22], the authors designed a motor with high torque density and low torque ripple by varying the interpole iron width for the rotor. Dae-Sung Jung et al. analyzed the characteristics of the permanent magnet type claw pole stepping motor by using 3D finite element analysis, selecting the teeth shape, the number of turns, and the PM overhang as the design factors [23]. In [24,25,26,27], the shape of the claw tooth was designed to analyze the influence of different shape parameters on motor performance. It was found that the tooth magnetic density is obviously affected by the height of the claw pole and the width of claw heel, but the air gap is not taken into account. In [28], the authors calculated the torque characteristics of the motor, such as the detent and the holding torques, and the step-position error by changing the gap between the upper and the lower stators and the staggered angle between the two stators. In [29], the authors found that cutting permanent magnet bearings has a positive effect on reducing braking torque in the design of a rotor. Furthermore, an improved holding torque waveform design model is proposed, which makes the holding torque waveform sinusoidal when the detent torque is reduced. In addition to using 3D finite element simulation, some scholars also use the equivalent magnetic circuit and finite element methods to analyze the torque characteristics of the motor [30,31]. Qun Jing Wang, Zheng Li, and Youyuan Ni et al. developed a model of a magnetic field inside the permanent magnet spherical stepper motor by using the integral equation method, and also derived discrete expressions for field distribution. However, the calculation error was too large to apply to the field of lens focusing [32]. Ioana Ionică et al. [33] studied the redundancy solution in order to maximize motor construction advantages. They found that alongside other parameters, the air gap thickness and the materials strongly influence the hybrid stepper motor design outcome. If the Hybrid stepper motor (HSM) air gap is increase by 25%, the torque will decrease by approximately 34%, but the optimization scheme of torque distortion caused by the high magnetic density of tooth when the air gap is small is not given. Dae-Sung Jung et al. [34] proposed a magnetic equivalent circuit considering axial flux to reduce the design time of a claw pole motor. Jae-Han Sim et al. [35] proposed an infinitesimal hexahedron-element-based 3D equivalent magnetic circuit network method in order to estimate the performance of the PM-assisted claw pole synchronous motor. In addition, some scholars [36,37] have analyzed the characteristics of the permanent magnet materials used in motors. Considering that many magnetic circuit equations are based on the assumption that the default permanent magnet is completely magnetized, but the actual permanent magnet is not completely magnetized [38], by analyzing the magnetization process, the magnetic properties of incompletely magnetized magnets were studied. When the motor is running, the current generates a magnetic field and the permanent magnet’s magnetic fields interact with each other. In [39], the authors analyzed the armature voltage, current waveform and harmonic content for different topologies.

In order to analyze the torque characteristics of the claw pole stepping motor, some of the above scholars used the 3D finite element method (FEM) to optimize the motor parameters, some used the equivalent magnetic circuit method to optimize the motor, and some scholars combined the finite element method with the equivalent magnetic circuit method. Although the FEM has high precision, the optimization time is long and the motor development cycle is prolonged. Although the equivalent magnetic circuit method can achieve results quickly, the calculation accuracy is not high, and so it may not be suitable for high-precision research. Based on the previous studies, this paper analyzes the electromagnetic field of a kind of focusing micro-claw pole permanent magnet stepper motor by using the FEM and design of experiment (DOE) method, and the correctness of simulation results is verified by experimental tests. Using the Taguchi method and optimization analysis method at the same time through experimental design can greatly reduce the time taken for optimization calculation. The influence of different design factors on the detent torque and holding torque of the motor is compared through the DOE method, and the design parameters that can reduce the detent torque and increase the holding torque of the micro-claw pole permanent magnet stepper motor are obtained through optimization analysis.

## 2. Structure of a Micro-Claw Pole Stepper Motor

The volume of the micro-claw pole stepper motor is very small, with a diameter of only 5 mm. Due to the limitation of the amount of rotor magnetization, the common step angles of micro-claw stepper motors are 9${}^{\circ }$ and 18${}^{\circ }$. The more the rotor is magnetized, the smaller the step angle is. A permanent magnet rotor with a step angle of 9${}^{\circ }$ has 20 poles, while the rotor of a stepping motor with a step angle of 18${}^{\circ }$ has 10 poles. A motor rotor adopting NdFeB-N52 permanent magnet material can possess a higher surface magnetic density. In the 2-2 phase excitation mode, the two phase coils are energized at the same time, and the torque ripple of the motor can be reduced by micro-steep drive. Air gap, claw height, claw thickness and permanent magnet thickness are the key parameters of the motor, and are also the objects of numerical optimization in this paper. The basic parameters of the motor studied in this paper are shown in Table 1.

**Figure 1:**
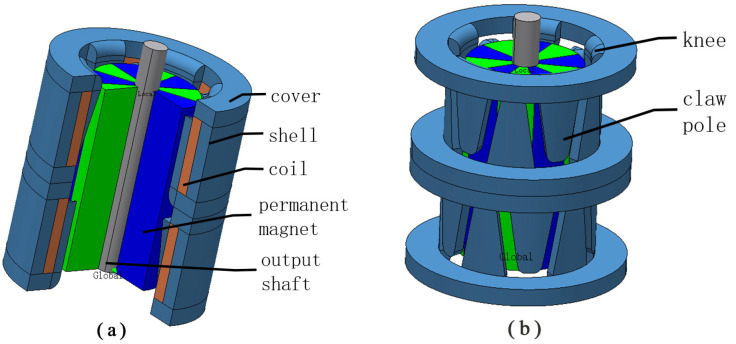
Structure diagram of a micro-claw permanent magnet stepper motor. (**a**) Overall schematic diagram of the motor. (**b**) The stator and permanent magnet rotor diagram of the motor.

The micro-claw pole permanent magnet stepping motor is composed of a permanent magnet rotor and stator, as shown in Figure 1. The stator consists of two covers, a shell, twenty claw poles, twenty knees and two coils. The rotor of the motor is composed of a permanent magnet and an output shaft. The claw pole is divided into two sections along the axial direction, and a 1/4 tooth pitch (90 electrical angle) is staggered between the two sections. Each section is composed of 10 claw poles meshing up and down, and all of them are wound with coils to form A, B two-phase winding. In the actual manufacture of claw poles, the knees are covered by a piece of iron sheet stamping manufacturing. The number of poles in the permanent magnet rotor is the same as the number of claw poles in the stator of each phase. By applying a pulse to the two-phase winding, the stepping motor is driven.

In this paper, three groups of air gap, claw pole height, claw pole thickness and permanent magnet thickness are selected at different levels, and then the Taguchi experiment method is used to carry out $L_{9}\left[3^{4}\right]$ orthogonal experiment on these four factors. The simulation results of holding torque and detent torque with 9 groups of different factor levels were obtained, and then the SNR was calculated for different factor levels and results. The influence degree of these four factors on holding torque and detent torque were obtained. Then, using the optimization analysis method, the four factors are numerically optimized. After 24 simulation tests, the parameter values that can increase the holding torque and decrease the detent torque were obtained. In order to ensure the correctness of the optimization simulation, this paper tests the holding torque value of the motor, and proves the correctness of the simulation optimization by comparing the test value with the simulation value.

## 3. Torque Analysis of Micro-Claw Pole Stepper Motor

**Figure 2:**
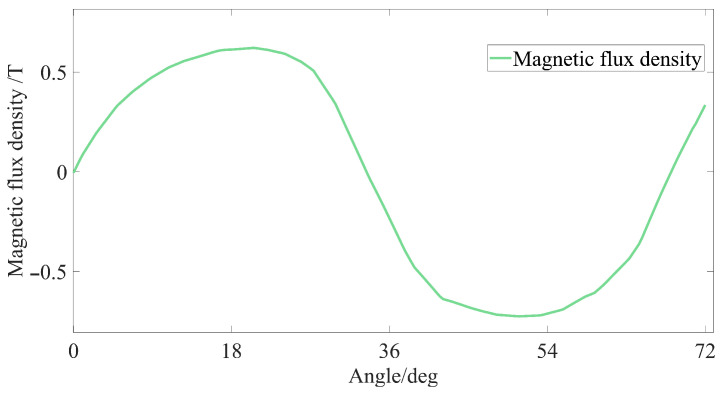
The air gap flux density waveform of the motor.

The detent torque of the micro-claw pole permanent magnet stepper motor is mainly affected by the structure of the claw pole and the permanent magnet. In the case that the stator coil is not energized, due to the magnetization of the permanent magnet, the detent torque will be generated between the claw pole and the permanent magnet. As shown in Figure 2, the shape of the claw poles is irregular, and the coupling area between the claw poles and the permanent magnets changes along the axial direction, resulting in sinusoidal distortion of the air gap flux density between the stator claw poles and the permanent magnets of the stepper motor. The torque fluctuation in the rotation process of the stepper motor is not conducive to the stable operation of the motor and adversely affects the control accuracy of the motor.

**Figure 3:**
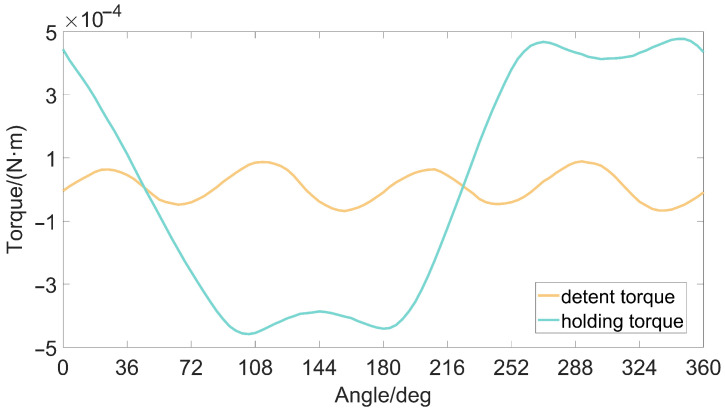
The holding torque and detent torque of the stepper motor.

The holding torque of the permanent magnet stepper motor is the maximum torque that the motor can achieve when the motor is powered on, that is, the peak value of the motor torque angle curve. In the motor application, the holding torque reflects the robustness of the motor and the ability to resist external disturbances. Its value is not only related to the body parameters of the motor, but also related to the current of the motor coil. The waveforms of the holding torque and the detent torque of the motor are shown in Figure 3. The period of the holding torque is a 360° electrical angle, and the period of the detent torque is a 90° electrical angle.

The commonly used optimization analysis methods for stepper motors include the analytical method, field circuit combination method, and electromagnetic field finite element analytical method. The analytical method has high efficiency but low calculation accuracy, and the empirical parameters have a great influence on the calculation results [10]. For a micro-claw pole permanent magnet stepper motor with an outer diameter of about 5 mm, the results calculated by the analytical method are more stringent.

Relying on the selection of empirical parameters, the FEM can be used to obtain accurate calculation results. In the calculation process, the calculation accuracy can be improved by setting a reasonable mesh density. At the same time, the experimental analysis method can be used to greatly reduce the amount of motor optimization and save time for optimization design.

When the current is constant, the torque of the motor is expressed by magnetic energy:

(1) $$T=\frac{dW_{c}}{d\theta }{|}_{i=constant}$$

(2) $$W_{c}=\frac{1}{2}\Lambda F^{2}$$

Equations (1) and (2) can be combined to obtain:

(3) $$T=\frac{dW_{c}}{d\theta }=\frac{1}{2}F^{2}\frac{d\Lambda }{d\theta }$$

In the equation above, *T* represents the torque of the motor when the current is constant, $W_{c}$ represents the magnetic common energy of the motor, θ represents the angle of motor motion, Λ represents the magnetic conductance of the magnetic flux, and *F* represents the magnetomotive force of the motor.

When there is no current, the formula of detent torque is as follows:

(4) $$T=\frac{1}{2}\Phi^{2}\frac{dR}{d\theta }$$

*R* represents the total reluctance of the magnetic flux, Φ represents the magnetic flux, and θ represents the angle that the motor turns.

Period of detent torque (electrical angle):

(5) $$C=K\times \frac{360^{\circ }}{LCM_{\left(N_{PM},N_{Claw}\right)}}$$

In the formula, *K* represents the number of pole pairs of the permanent magnet, $N_{Claw}$ represents the number of two-phase claw poles of the motor, and $LCM_{\left(N_{PM},N_{Claw}\right)}$ represents the least common multiple (LCM) between the number of magnetic poles of the permanent magnet and the number of poles of the motor claws. In this motor, the permanent magnet has 10 poles, and the two-phase coil has 20 claw poles. The calculated detent torque cycle is 90°.

Based on the finite element simulation calculation, this paper adopts the Taguchi method of DOE method to analyze the influence of different factors on the detent torque and holding torque of the micro-claw pole permanent magnet stepper motor. There is mutual influence between these parameters. For example, when the thickness of the magnet changes, the air gap between the stator and the rotor also changes. When the height of the magnet changes, the height of the claw pole also changes. Therefore, when optimizing the detent torque and the holding torque, it is necessary to consider the role of these parameters as a whole.

## 4. Parameter Optimization of Micro-Claw Pole Stepper Motor

The original parameters of the motor are shown as reference value 1 in Table 2. In this paper, reference values 2 and 3 are selected without changing the outer diameter and output shaft diameter of the motor. In this experiment, consisting of four factors and three levels, if the full factorial experiment method is used, a total of 81 experiments are required. In order to save time and reduce the number of experiments, this paper adopts the Taguchi method of partial factorial design to analyze each factor, and therefore only requires nine experiments using the following orthogonal experiment table $L_{9}\left[3^{4}\right]$. The positioning and holding torque peak values of the micro stepper motor at a speed of 800pps are calculated through nine quadrature experiments, as shown in Table 3.

The SNR of each factor to the response is obtained by the Taguchi analysis method, and the SNR measures the change in the response relative to the nominal value or target value under different noise conditions. The SNR is used as the quality index. The larger the value of the SNR, the better the signal quality.

In the Taguchi experiment, when it is desirable for the response to be as small as possible, the SNR can be calculated by the following formula:

(6) $$S/N=-10Log\frac{1}{n}\Sigma {y_{i}}^{2}$$

When it is desirable for the response to be as large as possible, the SNR can be calculated by the following formula:

(7) $$S/N=-10Log\frac{1}{n}\Sigma \frac{1}{y_{i}^{2}}$$

In the equation above, S/N is expressed as log times 10, and the unit of S/N is decibels (dB). The higher S/N is, the higher the quality of the response. *S* represents the response signal, *N* represents the noise signal, *n* represents the number of measurements, and $y_{i}$ is the error of the ith response.

**Figure 4:**
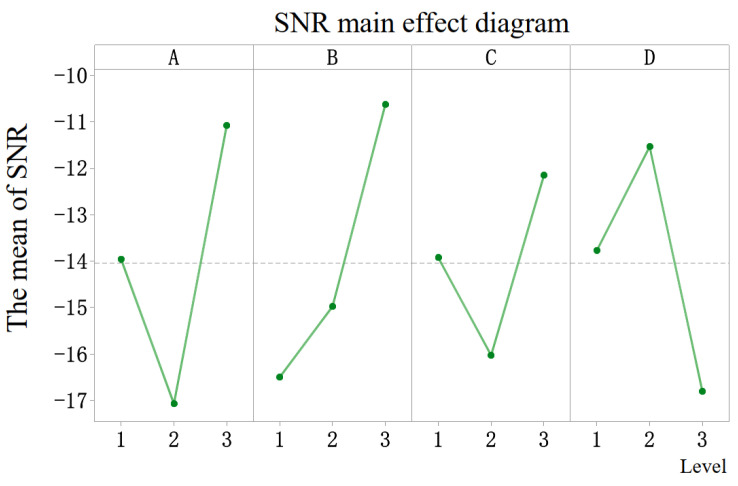
SNR diagram of each factor’s response to detent torque.

**Figure 5:**
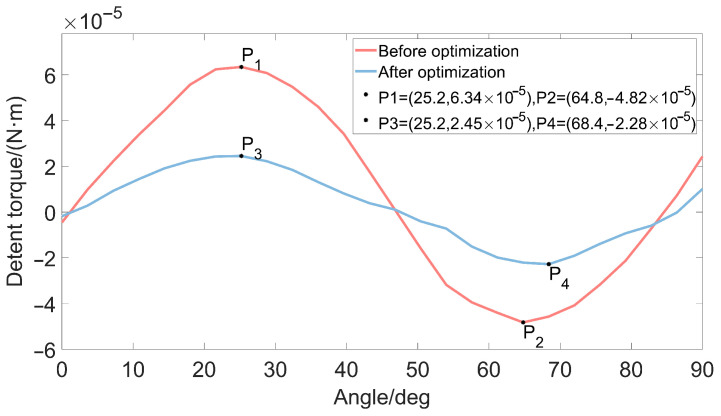
Comparison of detent torque before and after Taguchi analysis.

Table 4 and Table 5 show the calculated SNR of each factor to the detent torque and holding torque response. According to the rank order of the table, it can be seen that the air gap is the most important factor affecting the detent torque. The influence of the permanent magnet thickness on the detent torque is greater than that of the claw pole thickness on the detent torque, and the influence of the claw pole height on the detent torque is slightly weaker than the first three factors. As shown in Table 5, the order of the influence of these factors on the holding torque is magnet thickness > air gap > claw pole height > claw pole thickness. From the SNR diagram of each factor to the response in Figure 4, it is easy to see that the combination of each factor level that minimizes the detent torque is *g*3, $l_{p}$3, $h_{c}$3, $l_{c}$2. Figure 5 provides a comparison diagram of the detent torque waveform under this combination and the detent torque waveform before optimization. The period of the detent torque is a 90° electrical angle, which is consistent with the calculation result of Equation (5). As shown in Figure 5, the peak value of motor detent torque decreased by 57.6% after Taguchi analysis.

The larger the air gap, the smaller the detent torque value of the claw pole stepper motor. When the air gap increases, the air gap magnetic field density of the motor weakens again. The air gap magnetic field acts as a medium for electromechanical energy exchange. When the air gap magnetic density decreases, the effective torque of the motor will be weakened, so the holding torque of the motor will also be reduced. The detent torque of the claw pole stepper motor cannot be reduced only by increasing the air gap; the effect of the air gap on the holding torque also needs to be considered.

The magnet thickness of the claw stepping motor is the second most important factor affecting the detent torque, after the air gap. Under the same air gap, the larger the magnet thickness, the greater the detent torque. According to the result of the Taguchi analysis of SNR, for the micro-claw pole permanent magnet stepper motor, the influence of the magnet thickness on the holding torque is greater than that of the air gap on the holding torque. The air gap flux density increases rapidly as the thickness of the magnet increases. However, as the thickness of the magnet increases, the core loss, moment of inertia and cost of the motor also increase.

The influence of the claw pole thickness on the detent torque of the micro-claw pole stepper motor is slightly weaker than that of the air gap and the magnet thickness. The thinner the claw pole, the greater the magnetic drag when the magnetic flux passes through the claw pole. The more easily the magnetic path of the claw pole is saturated, the easier it is to generate leakage magnetic flux, and the smaller the magnetic flux through the main magnetic circuit, which weakens the ability of the motor to generate electromagnetic torque. However, the claw pole cannot be too thick. When the claw pole design is too thick, although the saturation of the claw pole magnetic circuit is avoided, the increase in the thickness of the claw pole reduces the cross-section width of the motor coil under the condition that the outer diameter of the motor is unchanged. The reduction in the number of coil turns will directly reduce the electromagnetic torque of the motor, so it will not be worth the loss when the detent torque is reduced by simply increasing the thickness of the claw pole.

The influence of the height of the claw pole on the motor detent torque is relatively small, but its influence on the holding torque is greater than that of the thickness of the claw pole on the holding torque. Because the magnetic flux at the knee of the claw pole passes through the claw pole, most of the magnetic flux will flow to the air gap through the root of the claw pole to form a magnetic circuit. The closer to the top of the claw pole, the less the magnetic flux, so the height of the claw pole has relatively little effect on the torque. In the design of the claw pole, the most appropriate ratio of the length of the root and the top of the claw pole is generally selected to achieve the best torque.

**Figure 6:**
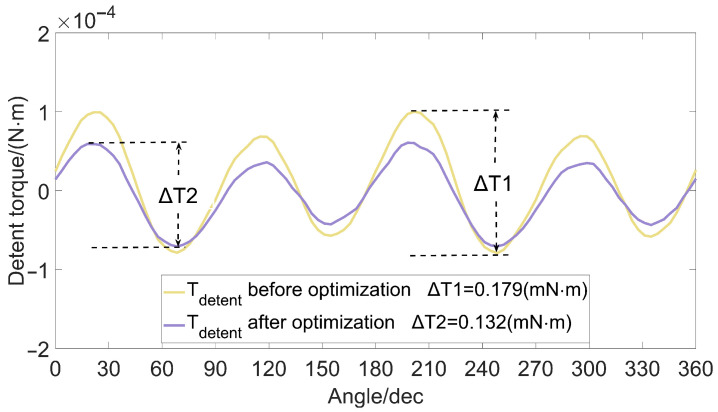
Comparison of detent torque.

**Figure 7:**
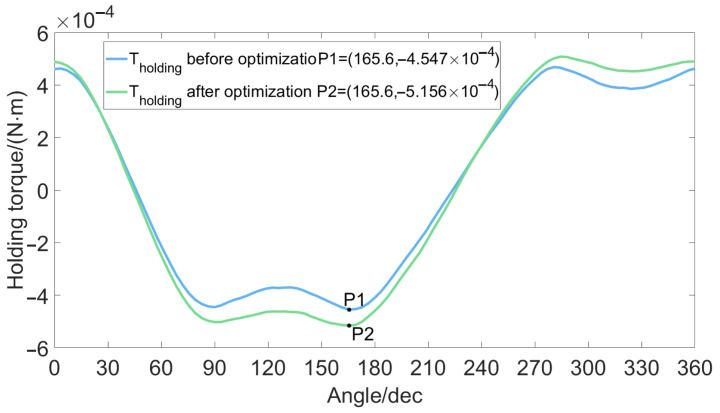
Comparison of holding torque.

While the detent torque is reduced, we also hope to increase the holding torque of the micro-claw pole stepper motor, thereby increasing the effective torque of the motor. In order to achieve this goal, this paper uses the optimization analysis method of experimental design to obtain a set of combination schemes for the air gap, magnet thickness, claw pole thickness, and claw pole height parameters of the miniature claw pole permanent magnet stepper motor, as shown in Table 6. The torque comparison before and after the optimization analysis is shown in Figure 6 and Figure 7. It is concluded that the detent torque of the optimal factor level is much smaller than the detent torque of each factor level before optimization, and the peak-to-peak value of the detent torque is reduced by 26.74%. The value of the holding torque is increased by 18.35%.

**Figure 8:**
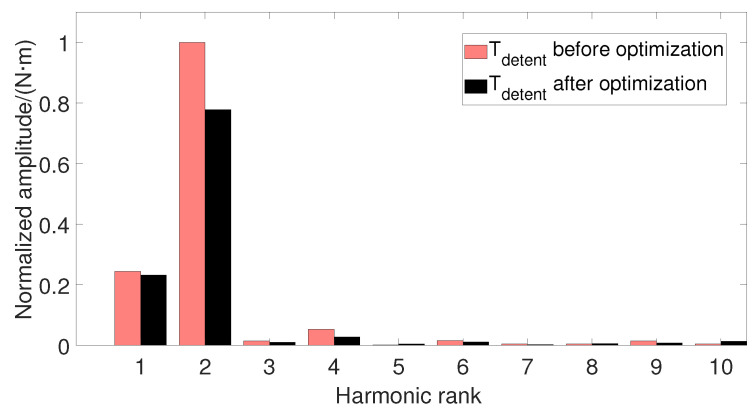
Comparison of detent torque harmonics before and after optimization.

The harmonic calculation of the detent torque before and after optimization is shown in Figure 8 below. It is found that the detent torque is mainly composed of the fundamental wave, the second harmonic and the 4th harmonic, and the amplitudes of other higher harmonics are close to zero, of which the second harmonic is the main factor. After optimization, the amplitude of the fundamental wave is slightly reduced, and the amplitude of the second harmonic is reduced by 22.2%.

**Figure 9:**
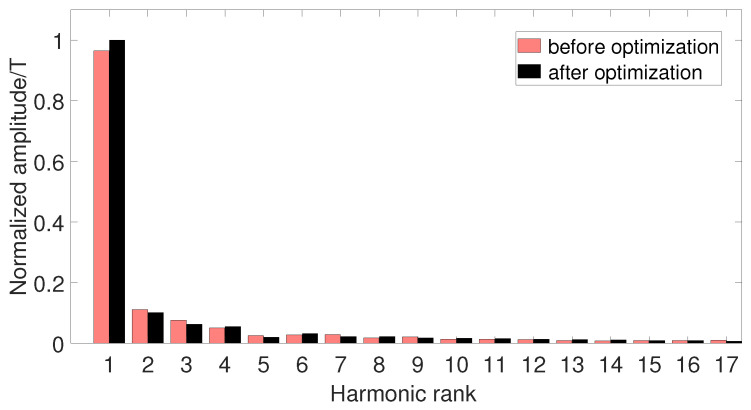
Comparison of the air gap flux density harmonics before and after optimization.

The no-load air-gap flux density of the motor is an important indicator for evaluating the performance of the motor. The harmonic analysis and comparison diagram of the air-gap flux density of the motor after this optimization design is shown in Figure 9. In the air gap magnetic density spectrum, the fundamental wave and the 4th harmonic exhibit a small increase, the 2th harmonic and the 3th harmonic exhibit a small decrease, and the amplitudes of other high-order harmonic components are basically unchanged. After optimization, the distortion rate of the air-gap flux density decreases from 16.45% to 14.81%.

## 5. Verification of Simulation Results

**Figure 10:**
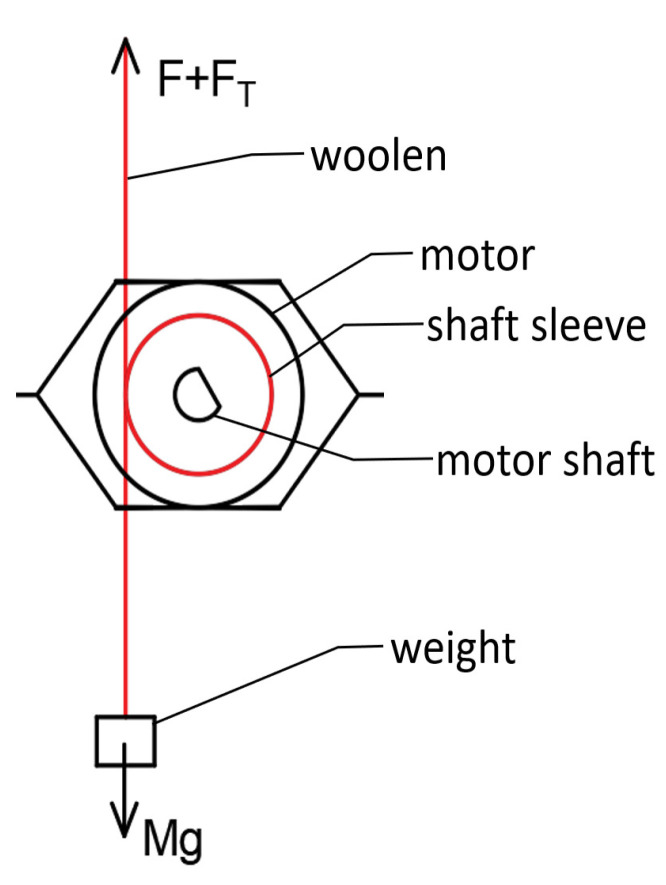
Schematic diagram of torque test bench.

In this paper, the holding torque of the micro-claw pole stepper motor is tested. By comparing and verifying the consistency between the simulation value and the test value of the holding torque before optimization, we illustrate the correctness of the simulation value of the stepper motor detent torque and holding torque optimization by the finite element method. A schematic diagram of the torque test is shown in Figure 10.

As shown in Figure 10 above, when the weight of *M* is just enough to “lock” the motor shaft sleeve, the following situation exists:

(1) If the direction of friction between the thin line and the shaft sleeve is upward along the thin line, then:

(8) $$Mg=F+F_{T}+f$$

(9) $$F_{T}=Mg-F-f$$

(2) If the direction of friction between the thin line and the shaft sleeve is downward along the thin line, then:

(10) $$Mg+f=F+F_{T}$$

(11) $$F_{T}=Mg-F+f$$

In the equation above, *M* is the mass of the weight, *F* is the reading of the spring scale, $F_{T}$ represents the tension corresponding to the torque of the motor, and *f* represents the friction between the thin line and the bushing. Based on the above four expressions, it can be seen that:

(12) $$F_{T}=Mg-F\pm f$$

When the static friction force is ignored during the test, the tension corresponding to the torque of the motor is:

(13) $$F_{T}=Mg-F$$

The formula of torque during the test is therefore:

(14) $$T=\frac{1}{2}\left(D+d\right)\times \left(Mg-F\right)\times 10^{-6}\left(N\cdot m\right)$$

where *T*/(N · m) represents the measured torque value of the motor, *D*/mm represents the diameter of the motor output shaft sleeve, *d*/mm represents the thin line diameter, *M*/g represents the reading of the spring scale and g=10 N/kg.

As shown in Table 7, the test average value of the holding torque is 0.497 (mN·m). Furthermore, the simulation value is 0.489 (mN·m). The tested holding torque value is similar to the simulation value, which verifies the correctness of the above simulation results.

**Figure 11:**
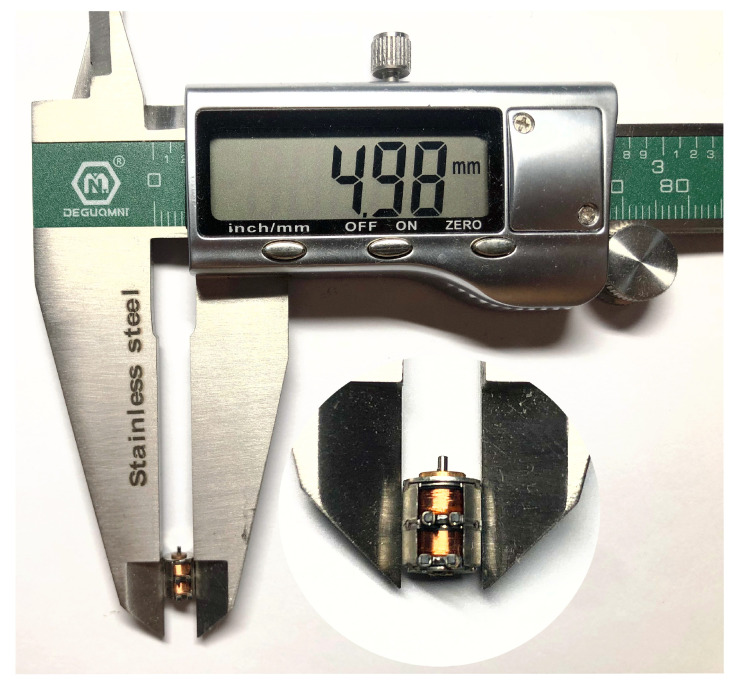
Photo of a 5 mm stepper motor.

**Figure 12:**
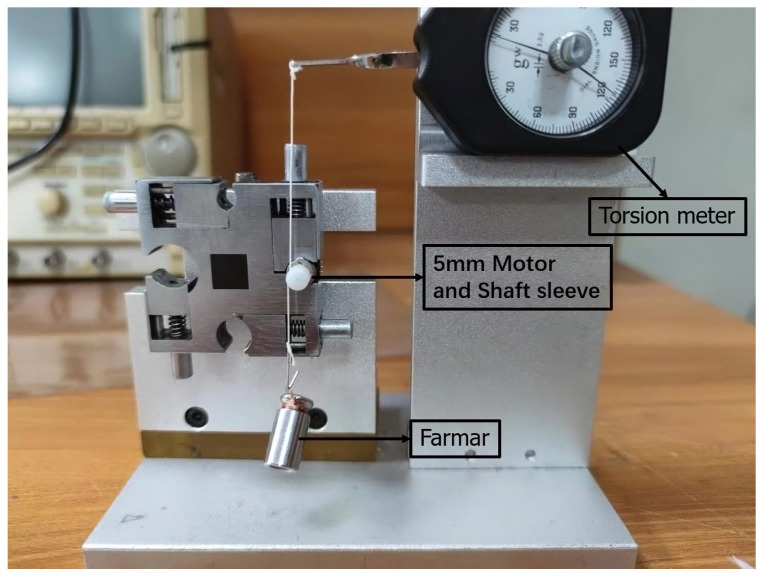
Micro stepper motor torque test bench.

Figure 11 shows a photo of the motor with a diameter of 5 mm (0.02 mm measurement error), and Figure 12 shows a photo of the motor test bench. As can be seen in Figure 12, the thin line is wound around the axle sleeve of the motor, one end is connected to the weight, the other end is connected to the spring scale, and there is a drive behind the test stand. During the test, the PWM constant-current chopper driving method was adopted, the test voltage was 3.3 V, and the current was limited to 200 mA. The single-phase resistance of the motor is 15 Ω, and during measurement, the motor produces a large amount of heat, rapidly affecting the measurement result of the motor holding torque. Thus, after every measurement, 3–5 s after the power is activated, 2 min should then pass before the next measurement.

## 6. Conclusions

In this paper, the FEM is used to carry out the 3D simulation of a micro-claw pole permanent magnet stepper motor. The responses of different factors to the detent torque and holding torque of a micro-claw pole permanent magnet stepper motor were analyzed by the DOE method. The degree of influence of different parameters on the response was obtained by calculating SNR. The results show that:

1. The air gap of the micro-claw pole permanent magnet stepper motor is the primary parameter affecting the detent torque, but it is a secondary factor affecting the holding torque. Both the detent torque and the holding torque are inversely proportional to the air gap;
2. The effect of the magnet thickness on the holding torque is greater than that of the air gap on the holding torque. The influence of the magnet thickness on the detent torque is greater than that of the claw pole thickness on the detent torque;
3. The height of the claw pole has little influence on the detent torque, and its influence on the holding torque is greater than that of the thickness of the claw pole on the holding torque. The height of the claw pole determines the axial length of the claw pole stepper motor. When the outer diameter of the motor is determined, we can optimize the height of the claw pole to compensate for the insufficient number of turns of the excitation coil;
4. In order to suppress the detent torque and increase the holding torque, if the air gap is not greatly increased, the thickness of the claw pole can be increased or the thickness of the permanent magnet can be reduced, but this also requires other parameters to match optimization.

Finally, the factor parameters for reducing the detent torque and increasing the holding torque are obtained by the experimental method of optimization analysis. The holding torque of a 5 mm claw pole stepper motor is tested and compared with the simulation value, which verifies the correctness of the simulation value. This provides a reference value for the further design and application of a micro-claw pole permanent magnet stepper motor.

## Figures and Tables

**Table 1:** Basic parameters of micro-claw pole.

Parameter	Value
Diameter *D*/mm	5
Step angle $\theta {/}^{\circ }$	18
Pole number	20
Drive method	2-2
Remanence $B_{r}$/T	1.45
Coercive force $H_{c}$/(KA/m)	1109
Air gap *g*/mm	0.15
Claw pole height $h_{c}$/mm	1.75
Claw pole thickness $l_{c}$/mm	0.27
Magnet thickness $l_{p}$/mm	1.05

**Table 2:** Reference values of different factors.

Reference	Air Gap g/mm	Magnet Thickness $l_{p}/$mm	Claw Pole Height $h_{c}/$mm	Claw Pole Thickness $l_{c}/$mm
1	0.15	1.05	1.75	0.27
2	0.13	0.96	1.85	0.30
3	0.17	0.93	1.60	0.25

**Table 3:** $L_{9}\left[3^{4}\right]$ orthogonal array table.

Experiment Number	Air Gap g/mm	Magnet Thickness $l_{p}$/mm	Claw Pole Height $h_{c}/$mm	Claw Pole Thickness $l_{c}$/mm	Detent Torque $Y\times 10^{-5}/$(N · m)	Holding Torque $Y\times 10^{-4}/$(N · m)
1	1	1	1	1	6.34	4.77
2	1	2	2	2	5.24	4.57
3	1	3	3	3	3.73	3.95
4	2	1	2	3	16.4	4.98
5	2	2	3	1	6.21	4.27
6	2	3	1	2	3.57	4.19
7	3	1	3	2	2.87	4.53
8	3	2	1	3	5.42	3.98
9	3	3	2	1	2.95	3.84

**Table 4:** SNR response table of detent torque.

Level	Air Gap (*g*)	Magnet Thickness ($l_{p}$)	Claw Pole Height ($h_{c}$)	Claw Pole Thickness ($l_{c}$)
1	−13.95	−16.50	−13.93	−13.77
2	−17.07	−14.98	−16.03	−11.53
3	−11.08	−10.63	−12.15	−16.80
Delta	5.99	5.87	3.88	5.27
Rank	1	2	4	3

**Table 5:** SNR response table of holding torque.

Level	Air Gap (*g*)	Magnet Thickness ($l_{p}$)	Claw Pole Height ($h_{c}$)	Claw Pole Thickness ($l_{c}$)
1	12.90	13.55	12.67	12.62
2	13.00	12.60	12.94	12.92
3	12.27	12.02	12.55	12.62
Delta	0.73	1.52	0.39	0.30
Rank	2	1	3	4

**Table 6:** Optimization analysis parameters.

Air Gap (*g*)	Magnet Thickness ($l_{p}$)	Claw Pole Height ($h_{c}$)	Claw Pole Thickness ($l_{c}$)	Holding Torque Fit Value N/m	Detent Torque Fit Value N/m
0.141	1.070	1.776	0.301	5.048$\;\times \;10^{-4}$	5.663$\;\times \;10^{-5}$

**Table 7:** Stepper motor holding torque test values.

Test Current I/A	Arm L/mm	Test Value F/N	Holding Torque T/(N · m)
0.2	2.01	0.227	4.56$\;\times \;10^{-4}$
0.2	2.01	0.264	5.31$\;\times \;10^{-4}$
0.2	2.01	0.228	4.58$\;\times \;10^{-4}$
0.2	2.01	0.254	5.11$\;\times \;10^{-4}$
0.2	2.01	0.262	5.27$\;\times \;10^{-4}$

## Abbreviations

The following abbreviations are used in this manuscript:

SNR	Signal-to-noise ratio
HSM	Hybrid stepper motor
FEM	Finite element method
DOE	Design of experiment
LCM	Least common multiple
